# Author Response Letter: “Correspondence: Is there an association between centre volume and survival or neurological outcomes among out-of-hospital cardiac arrest patients?”

**DOI:** 10.1186/s12873-022-00744-z

**Published:** 2022-12-11

**Authors:** Takumi Tsuchida, Mineji Hayakawa

**Affiliations:** Department of Emergency Medicine, Hokkaido University Hospital, N14W5 Kita-ku, Sapporo, 060-8648 Japan

**Keywords:** Cardiopulmonary resuscitation, Hospital volume, Neurological outcome, Out-of-hospital cardiac arrest, Prediction, Prognosis

## Comment

We thank Goh and Ho for sharing opinions that enhanced the content of our paper. Based on their comments, we present discussions that will be applicable to the future research.

As per Goh et al. reviews, the conflicting results between our study and the previous studies can be explained, by stating the fact that there are no differences in hospital characteristics other than the number of out-of-hospital cardiac arrest (OHCA) patients delivered [1]. As we reported, in Japan; most of the hospitals that received transferred OHCA patients have specialists in each department and can perform extracorporeal membrane oxygenation (ECMO) [1]. Although not shown in the paper, all of these hospitals have 24/7 availability to percutaneous coronary intervention (PCI), and almost all of them perform therapeutic temperature management (TTM) (except one low-volume hospital and one middle-volume hospital). Probably, due to the presence of this background, there have been no reports from Japan showing a significant difference between hospital size and prognosis of OHCA patients [1–3]. One report, however, shows the advantage of Critical Care Medicine or Medical Centres (CCMC) over non-CCMC for the outcome of OHCA patients [3]. CCMC in Japan should be capable to provide advanced medical care on a 24-h basis, and advanced treatments such as PCI, TTM, and ECMO, the main focus in Goh et al.’s comments, are a standard practice. In our study, we see no significant correlation between hospital size and prognosis for patients with OHCA in an analysis limited to CCMC [1].

Goh et al. stated that post-cardiac arrest care, such as 24/7 PCI, TTM and ECMO capabilities, needs to be matched. However, in our study, these factors matched spontaneously. Therefore, our results were not affected by the availability factor of 24/7 PCI, TTM and ECMO. In our study, the neurological outcomes in patients with pre-hospital return of spontaneous circulation improved in high-volume hospitals. This result suggests that the hospital volume, i.e., experience in treating a large number of OHCA patients, also affects the neurological outcome of OHCA patients to some extent.

One major reason for the inconclusive results on this topic may be the varying degrees of centralization of hospital functions in different countries. Thus, recent systematic reviews and meta-analyses have also not reached certain conclusions [4, 5]. In fact, the heterogeneity of studies used in these systematic reviews and meta-analyses is high.

In areas where the concentration of advanced equipment in large facilities is high, facilities with a higher number of transferred OHCA patients have a better prognosis. To examine the impact of the number of genuine OHCA patient transfers on the prognosis of OHCA patients in such areas, it is necessary to match post-cardiac arrest care, such as 24/7 PCI, TTM and ECMO arrangements, as suggested by Goh et al.

We hope, we have clarified the queries to the best extent possible.

## Data Availability

The datasets used and/or analyzed during the current study are available from the corresponding author upon reasonable request.

## Abbreviations

CCMC: Critical care medicine or medical centres
ECMO: Extra corporeal membrane oxygenation
OHCA: Out-of-hospital cardiac arrest
PCI: Percutaneous coronary intervention
TTM: Temperature management
